# A Novel Decision Making Procedure during Wakefulness for Screening Obstructive Sleep Apnea using Anthropometric Information and Tracheal Breathing Sounds

**DOI:** 10.1038/s41598-019-47998-5

**Published:** 2019-08-07

**Authors:** Ahmed Elwali, Zahra Moussavi

**Affiliations:** 1 Biomedical Engineering, University of Manitoba, Winnipeg, Canada; 2 Electrical and Computer Engineering, University of Manitoba, Winnipeg, Canada

**Keywords:** Translational research, Biomedical engineering, Translational research, Biomedical engineering

## Abstract

Obstructive sleep apnea (OSA) is an underdiagnosed common disorder. Undiagnosed OSA, in particular, increases the perioperative morbidity and mortality risks for OSA patients undergoing surgery requiring full anesthesia. OSA screening using the gold standard, Polysomnography (PSG), is expensive and time-consuming. This study offers an objective and accurate tool for screening OSA during wakefulness by a few minutes of breathing sounds recording. Our proposed algorithm (AWakeOSA) extracts an optimized set (3–4) of breathing sound features specific to each anthropometric feature (i.e. age, sex, etc.) for each subject. These personalized group (e.g. age) classification features are then used to determine OSA severity in the test subject for that anthropomorphic parameter. Each of the anthropomorphic parameter classifications is weighted and summed to produce a final OSA severity classification. The tracheal breathing sounds of 199 individuals (109 with apnea/hypopnea index (AHI) < 15 as non-OSA and 90 with AHI ≥ 15 as moderate/severe-OSA) were recorded during wakefulness in the supine position. The sound features sensitive to OSA were extracted from a training set (n = 100). The rest were used as a blind test dataset. Using Random-Forest classification, the training dataset was shuffled 1200–6000 times to avoid any training bias. This routine resulted in 81.4%, 80.9%, and 82.1% classification accuracy, sensitivity, and specificity, respectively, on the blind-test dataset which was similar to the results for the out-of-bag-validation applied to the training dataset. These results provide a proof of concept for AWakeOSA algorithm as an accurate, reliable and quick OSA screening tool that can be done in less than 10 minutes during wakefulness.

## Introduction

Obstructive sleep apnea (OSA) is a common syndrome characterized by repetitive episodes of complete (apnea) or partial (hypopnea) pharyngeal collapse during sleep^1^. The severity of OSA is commonly measured by the apnea/hypopnea index (AHI), which is the number of apnea/hypopnea episodes per hour of sleep. Usually, an AHI < 5 is considered as non-OSA, 5 < AHI < 15 as mild, 15 < AHI < 30 as moderate and AHI > 30 as severe OSA. Clinically, however, it is a common practice to consider individuals with AHI < 15 as those who may not benefit from treatment, and therefore an AHI of 15 is used as a threshold to determine severity^2,3^. Signs and symptoms of OSA include excessive daytime sleepiness, loud snoring, and observed episodes of breathing ceasing, gasping and/or choking during sleep^4^. OSA can severely impact the quality of sleep, and therefore the quality of life. It is associated with an increased risk of developing cardiovascular problems, hypertension, stroke, depression, diabetes, and headaches, as well as traffic accidents^5^. These comorbidities may be worsened if OSA is not treated^6^. In addition, it has been suggested to consider the existence of critical comorbidity for OSA to determine its severity and its treatment management^7^. Furthermore, not taking suitable precautions (due to lack of accurate and reliable screening tools for OSA) prior to a full anesthesia of OSA patients undergoing a sugary may lead to perioperative morbidity and mortality^8,9^. An accurate OSA screening tool, in particular for patients prior to undergoing a surgery requiring a full anesthesia, would reduce these risks^8,9^. This paper reports on a novel OSA classification procedure as a quick and accurate screening tool, based on anthropometric information and a few minutes of breathing sounds recorded during wakefulness.

The gold standard for OSA diagnosis is an overnight Polysomnography (PSG) assessment. However, it is costly and time-consuming. There are many portable monitoring devices for OSA, but they all require an overnight recording. In Canada and US, about 10% of the population suffer from OSA^10^, while the number of qualified sleep rooms available for conducting PSG studies is limited. Consequently, there is a long waiting list of patients; in some places, the waiting time exceeds a year for an overnight full PSG. Due to the mentioned facts, it is very desirable for anesthesiologists, in particular, to have an efficient perioperative management plan based on an objective, reliable and prompt diagnostic or screening tool for OSA^8,9,11^.

A quick OSA screening tool that is commonly used for patients undergoing surgery requiring full anesthesia is the STOP-BANG questionnaire^12^. It is a simple, quick, and inexpensive assessment that is reported to have a high sensitivity (~93%) but at the cost of a very poor specificity (~36%)^12^. Any assessment with poor specificity indirectly increases the referral rate to the full PSG study; thus, increases healthcare system’s cost. Therefore, there is a need for a quick and reliable objective technology with high sensitivity and specificity for OSA screening applicable during wakefulness.

Our team has been pioneering in proposing the use of tracheal breathing sound analysis during wakefulness for screening OSA^13–16^. Aside from our team, several research groups around the globe have been working on the possibility of using either tracheal breathing or vocal sounds during wakefulness to predict OSA^17–20^. Overall, those studies have reported an accuracy between 79.8 to 90% with both comparable sensitivity and specificity. While their accuracy is much better than the STOP-Bang questionnaire, none of the reported accuracies indicate blind test accuracy. In addition, their unbalanced sample sizes were quite small [23 (minimum 10 subjects for non-OSA) and 70 (minimum 13 subjects for OSA)] given the heterogeneity of OSA population.

In our team, we have investigated the use of tracheal breathing sounds analysis for screening OSA. Breathing sounds recorded from the mouth and nose were first sequestered into inspiratory and expiratory sounds; then, the characteristic features were extracted by spectral, bispectral and fractal analyses, followed by a classification routine for estimating the severity of OSA.

Our hypothesis is that the upper airway (UA) deformities due to OSA affects the breathing sounds even during wakefulness, and that effect should be detectable by tracheal breathing sounds analysis^15,21,22^. In our team’s previous works^13–16^, we showed the proof of concept for this hypothesis. Moreover, in our recent work, we achieved a testing classification accuracy of ∼84% with comparable (<10% difference) specificity and sensitivity for two balanced groups of non-OSA (AHI ≤ 5, n = 61) and OSA (AHI ≥ 10, n = 69)^16^. We also showed a significant superiority of using tracheal breathing sound features over the use of only anthropometric information (i.e., sex, age, neck circumference) for screening OSA during wakefulness^16^. However, the effects of the anthropometric confounding variables (i.e., age, sex, height, etc.) on the sound signals were not investigated. In addition, as the goal is to have a quick screening with high sensitivity of identifying the OSA individuals in need of treatment, it is desirable to have only one threshold (e.g., AHI = 15) for such decision making. Although it is difficult to distinguish an AHI of 14 and 16 from each other, the AHI = 15 was chosen as the threshold because it is the most common clinically accepted threshold to separate OSA individuals in need of treatment from those who do not benefit from a treatment^2,3^. When we applied the technique used in our previous publication mentioned above with this threshold AHI = 15, the blind-test accuracy dropped to <70%, which is not desirable. It is commonly discussed that AHI is not the best indicator of a diagnostic decision for OSA. Sleep medicine doctors usually base their decision on several factors such as daytime sleepiness, number of arousals/night, etc. along with AHI. However, for a quick screening with automated real-time screening, we have to have a reference for accuracy, and AHI is the most common standard used. Therefore, we propose a new decision-making algorithm, called AWakeOSA, using anthropometric information and a few minutes of tracheal breathing sounds recorded during wakefulness to identify OSA individuals in need of treatment. This algorithm considers the confounding anthropometric effects on the breathing sounds and uses them in to predict the severity of OSA in a subgroup with a similar confounding variable.

**Figure 1:**
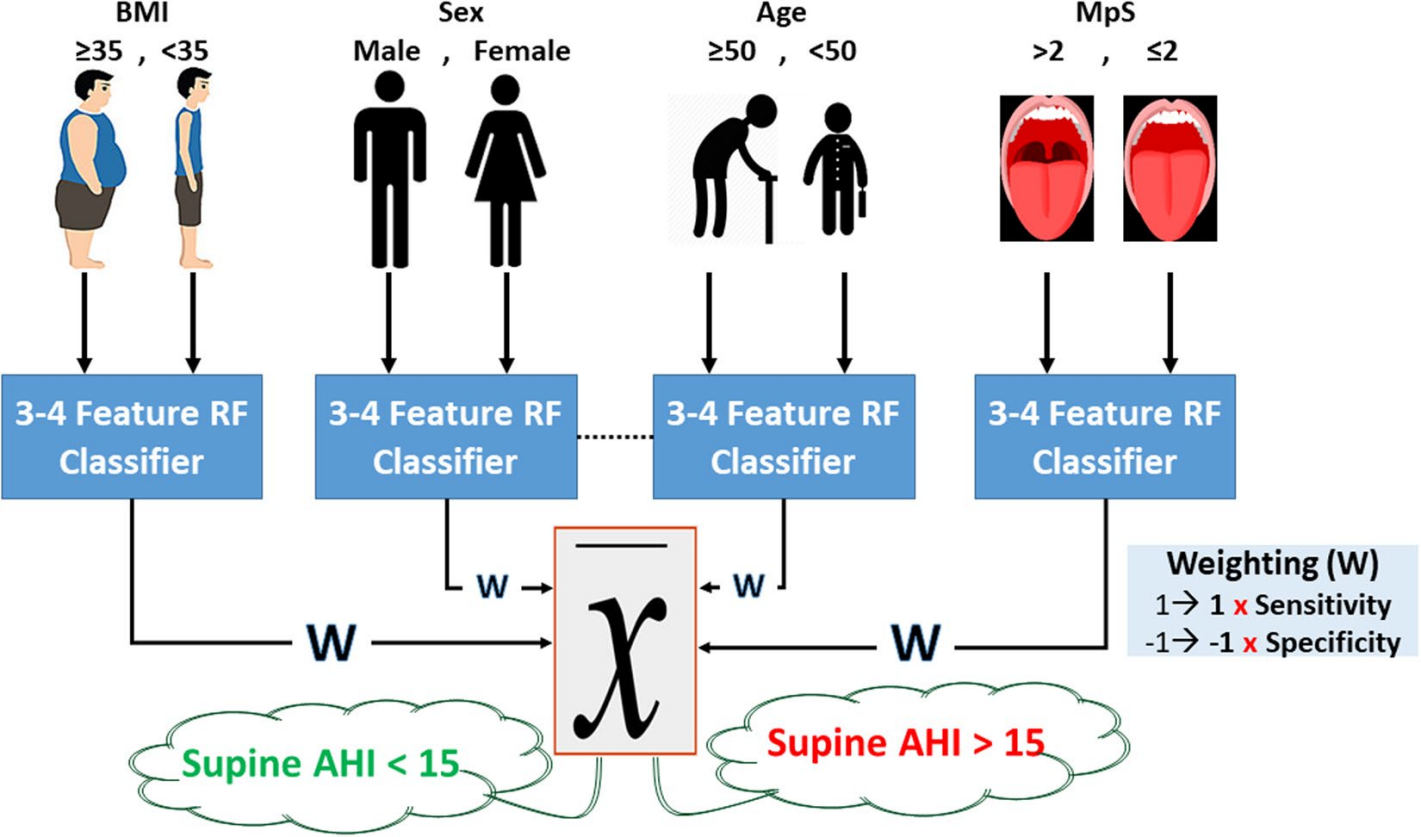
The AWakeOSA algorithm used for decision making routine using the weighted outcomes from each of the used anthropometric subsets classifiers. Legend: RF: random forest; AHI: apnea-hypopnea index.

The premise of the AWakeOSA algorithm is to find the best sound features sensitive to OSA severity (determined by AHI) for each subgroup of individuals with a specific anthropometric factor (i.e., age, sex, weight, etc.); the algorithm is shown schematically in Fig. 1. Since the OSA population is very heterogeneous and many confounding variables such as age, sex, height, weight, etc. affect breathing sound characteristics^23–26^, it is challenging to have some sound features predicting AHI for all individuals. The proposed AWakeOSA algorithm (Fig. 1) overcomes this challenge by grouping individuals into subgroups based on their specific anthropometric factors that in turn affect breathing sounds. Then, in each subgroup, the best sound features to predict AHI are extracted, and a classifier is trained using a training set of data. The classifiers’ outcomes in each subgroup are then used in a weighted average voting scheme to make the classification decision OSA (AHI > 15) or non-OSA (AHI < 15).

In this paper, we present the results of the AWakeOSA algorithm for an AHI = 15 as the threshold for data collected from 199 individuals with various severity of OSA (AHI was between 0 to 143), out of which about 45% of data were set aside as a blind test and the remaining was used for extracting features and training the classifiers. In all instances, we used a two-group classification algorithm based on the random forest algorithm^27^.

## Results

In this study, the breathing sounds data of 199 participants made up of 109 non-OSA individuals (50 males, AHI < 15) and 90 OSA individuals (66 males, AHI > 15) were used. All data were recorded during wakefulness in the evening (~8 PM) before the participants proceed to PSG sleep study. Anthropometric parameters for the two groups are reported in Table 1. The data of the two groups were not matched in terms of any of the confounding variables: sex (male/female), body mass index (BMI threshold = 35), neck circumference (NC threshold = 40), age (threshold = 50), or Mallampati score (MpS threshold = 3). Out of the 199 individuals’ dataset, 86 individuals (47 non-OSA and 39 OSA) data were set aside as a blind test for assessing the algorithm’s accuracy. Table 1 also shows the anthropometric information for the two groups of training and testing.

**Table 1:** Participants’ anthropometric information.

	AHI_Supine_	Age		Sex	BMI		NC		MpS
		n > 50	n ≤ 50		n ≥ 35	n < 35	n > 40	n ≤ 40	
**The entire dataset (199 subjects)**									
Non-OSA (AHI < 15, n = 109)	3.59 ± 3.95	48.60 ± 12.69		50 M, 59 F	31.79 ± 7.21		39.81 ± 5.14		59 ‘I’, 25 ‘II’, 15 ‘III’ and 9 ‘IV’
		50	59		28	81	50	59	
OSA (AHI ≥ 15, n = 90)	42.85 ± 32.72	52.18 ± 11.55		66 M, 24 F	36.44 ± 8.01		44.08 ± 3.67		22 ‘I’, 30 ‘II’, 22 ‘III’ and 16 ‘IV’
		52	38		44	46	72	18	
**The training dataset (113 subjects)**									
Non-OSA (AHI < 15, n = 62)	3.4 ± 3.69	49.18 ± 12.89		32 M, 30 F	31.69 ± 7.42		40.06 ± 5.22		32 ‘I’, 14 ‘II’, 11 ‘III’ and 4 ‘IV’
		30	32		18	44	32	30	
OSA (AHI ≥ 15, n = 51)	52.79 ± 39.91	51.96 ± 12.03		36 M, 15 F	37.33 ± 9.01		43.94 ± 3.85		13 ‘I’, 14 ‘II’, 16 ‘III’ and 8 ‘IV’
		29	22		25	26	39	9	
**The Blind testing dataset (86 subjects)**									
Non-OSA (AHI < 15, n = 47)	3.85 ± 4.29	47.83 ± 12.52		18 M, 29 F	31.92 ± 7.00		39.47 ± 5.05		27 ‘I’, 11 ‘II’, 4 ‘III’ and 5 ‘IV’
		20	27		10	37	18	26	
OSA (AHI ≥ 15, n = 39)	29.86 ± 17.63	52.46 ± 11.04		30 M, 9 F	35.26 ± 6.40		44.25 ± 3.48		9 ‘I’, 16 ‘II’, 6 ‘III’ and 8 ‘IV’
		23	16		19	20	33	6	

AHI: apnea-hypopnea index, BMI: body mass index, NC: neck circumference, MPS: mallampati score, M/F: male/female.

AHI was found to have significant correlations with BMI, NC, and MpS (r = 0.44, 0.43, and 0.26, respectively). However, when we used the available anthropometric information (i.e., BMI, age, NC, and sex) of the STOP-BANG questionnaire^12^ and classified the entire dataset (199 subjects) for the two OSA severity groups (with AHI = 15 as threshold), the resulted classification accuracy, specificity, and sensitivity were found to be only 63.4%, 74.3%, and 50.5%, respectively.

Next, we analyzed the recorded breathing sounds collected from four breathing maneuvers (i.e., Mouth and Nose –inspiration and -expiration). While we used a sharp threshold of AHI = 15 for training classifiers, for the feature extraction and reduction stage, only data of subjects with AHI ≤ 10 (n = 60) and AHI ≥ 20 (n = 40) in the training dataset were used. Using the algorithm shown in Fig. 1, we analyzed the recorded breathing sounds in each of the following subsets of the training dataset separately: BMI < 35, Age > 50, Age ≤ 50, male, NC > 40, and MpS ≤ 2. Subjects in each subset were matched with respect to only one anthropometric variable. We did not have subsets of BME > 35 or NC < 40, etc due to limited sample size. The selected and used subsets had ≥30 non-OSA subjects and ≥20 OSA subjects, which were reasonable numbers for feature extraction and reduction and group classification.

The number of breathing sound features extracted from the two breathing maneuvers, while analyzing inspiratory and expiratory phases separately, was around 250. Using the proposed feature reduction procedure (see appendix) on the training dataset, around 15 features per each anthropometric subset were selected for further investigation. These sound features showed significant differences (p < 0.05) between the two OSA severity groups as they were highly correlated (p < 0.01) with AHI. In addition, they showed an effect size >0.8. Table 2 shows the selected sound features’ definition and where they were extracted from, the subsets and the correlation coefficients of the selected sound features and AHI in the entire training dataset and each anthropometric subset.

**Table 2:** Descriptions and details of the selected features.

FN	BM	Feature’s definition	Subset	CC
1	ExpM	$$\begin{array}{c}f2=235\\ f1=130\end{array}\,Mean\,of\,P(f)-\begin{array}{c}f2=1410\\ f1=1260\end{array}\,Mean\,of\,P(f)$$	**All data**	−0.40
2	InsN	$$\begin{array}{c}f2=355\\ f1=250\end{array}\,Mean\,of\,the\,slope\,of\,P(f)$$	**All data**	0.37
3	ExpM	$$\begin{array}{c}f2=550\\ f1=300\end{array}\,Bandwidth\,of\,the\,spectral\,centroid\,of\,P(f)$$	**All data**	−0.48
4	InsM	$$\begin{array}{c}f2=1515\\ f1=1200\end{array}\,First\,order\,moment\,of\,the\,positive\,diagonal\,of\,B(f,f)$$	**All data**	0.25
5	InsM	$$\begin{array}{c}f2=270\\ f1=140\end{array}\,Gmean\,of\,P(f)$$	BMI < 35	−0.41
6	InsM	$$\begin{array}{c}f2=270\\ f1=140\end{array}\,First\,order\,moment\,of\,B(f,f)\,on\,0.5f-f\,line$$	BMI < 35	−0.45
7	InsN	$$\begin{array}{c}f2=275\\ f1=130\end{array}\,Mean\,of\,B(f,f)$$	BMI < 35	−0.40
8	InsN	$$\begin{array}{c}f2=max\\ f1=min\end{array}\,Weight\,center\,of\,the\,positive\,diagonal\,of\,B(f,f)$$	BMI < 35	0.43
9*	InsN	$$\begin{array}{c}f2=280\\ f1=130\end{array}\,Mean\,of\,P(f)$$	**Age** **>** **50**	−0.35
10*	InsN	$$\begin{array}{c}f2=560\\ f1=80\end{array}\,Spectral\,centroid\,of\,P(f)$$	**Age** **>** **50**	0.37
11	InsM	$$\begin{array}{c}f2=230\\ f1=130\end{array}\,Weight\,center\,of\,the\,positive\,diagonal\,of\,B(f,f)$$	**Age** **>** **50**	0.32
12	InsN	$$\begin{array}{c}f2=280\\ f1=130\end{array}\,Mean\,of\,B(f,f)$$	**Age** **>** **50**	−0.37
13	InsN	$$\begin{array}{c}f2=280\\ f1=130\end{array}\,Second\,order\,moment\,of\,the\,negative\,diagonal\,of\,B(f,f)$$	**Age** **>** **50**	−0.48
14	InsN	$$\begin{array}{c}f2=370\\ f1=270\end{array}\,Mean\,of\,the\,slope\,of\,P(f)$$	Age ≤ 50	0.52
15	ExpN	$$\begin{array}{c}f2=550\\ f1=450\end{array}\,Mean\,of\,the\,slope\,of\,P(f)$$	Age ≤ 50	−0.41
16	InsM	$$\begin{array}{c}f2=510\\ f1=390\end{array}\,Weight\,center\,of\,B(f,f)\,on\,0.5f-f\,line$$	Age ≤ 50	0.40
17	InsN	$$\begin{array}{c}f2=350\\ f1=250\end{array}\,Mean\,of\,the\,slope\,of\,P(f)$$	**Male**	0.42
18	InsN	$Frequency\,of\,the\,first\,peak\,for\,of\,P(f)\,with\,a\,cuttoff\,of\,550\,Hz$	**Male**	0.53
19	InsN	$$\begin{array}{c}f2=520\\ f1=395\end{array}\,Mean\,of\,P(f)$$	NC > 40	0.37
20	InsM	$$\begin{array}{c}f2=600\\ f1=60\end{array}\,Frequesncy\,of\,the\,average\,of\,Maximum\,peaks\,of\,P(f)$$	NC > 40	0.29
21	InsM	$$\begin{array}{c}f2=600\\ f1=60\end{array}\,Weight\,center\,of\,B(f,f)\,on\,f-2f\,line$$	NC > 40	0.35
22	InsN	$$\begin{array}{c}f2=520\\ f1=395\end{array}\,Weight\,center\,of\,the\,positive\,diagonal\,of\,B(f,f)$$	NC > 40	0.28
23	InsN	$$\begin{array}{c}f2=350\\ f1=250\end{array}\,Mean\,of\,the\,slope\,of\,P(f)$$	**MpS** **<** **3**	0.34
24	InsM	$$\begin{array}{c}f2=1460\\ f1=1090\end{array}\,Weight\,center\,of\,B(f,f)\,on\,f-2f\,line$$	**MpS** **<** **3**	0.27
25	InsM	$$\begin{array}{c}f2=1460\\ f1=1260\end{array}\,First\,order\,moment\,of\,B(f,f)\,on\,f-2f\,line$$	**MpS** **<** **3**	0.39
26	ExpM	$$\begin{array}{c}f2=1410\\ f1=1260\end{array}\,First\,order\,moment\,of\,the\,negative\,diagonal\,of\,B(f,f)$$	**MpS** **<** **3**	0.36

Legend: INS/EXP: inspiration/expiration, M/N: mouth/nose, mean: arithmetic mean, gmean: geometric mean, P(F): the power spectrum, B(F,F): the bispectrum, F: frequency, FN: feature number, BM: breathing maneuver, subset: subset of usage, BMI: body mass index, NC: neck circumference, MPS: mallampati score, CC: the correlation coefficient with AHI. All correlations were significant at *P* < *0.01* Level.

*Features 9 and 10 were used alternatively.

In the next stage of the AWakeOSA algorithm, we used the selected sound features and one anthropometric feature, the NC, as the features for classification because it showed a significant correlation (0.43, with p < 0.01) with AHI when tested on the training dataset. The NC feature was investigated further in all subsets other than its own subset. Thus, the selected sound features and NC were divided into three- and four-feature combinations. These feature combinations were used to classify each participant’s data in every subset to one of the two OSA severity classes. Table 2 column 4 shows the sound features used per anthropometric subset constructing the combination for classification. NC was selected and used in age ≤50 and male subsets.

**Figure 2:**
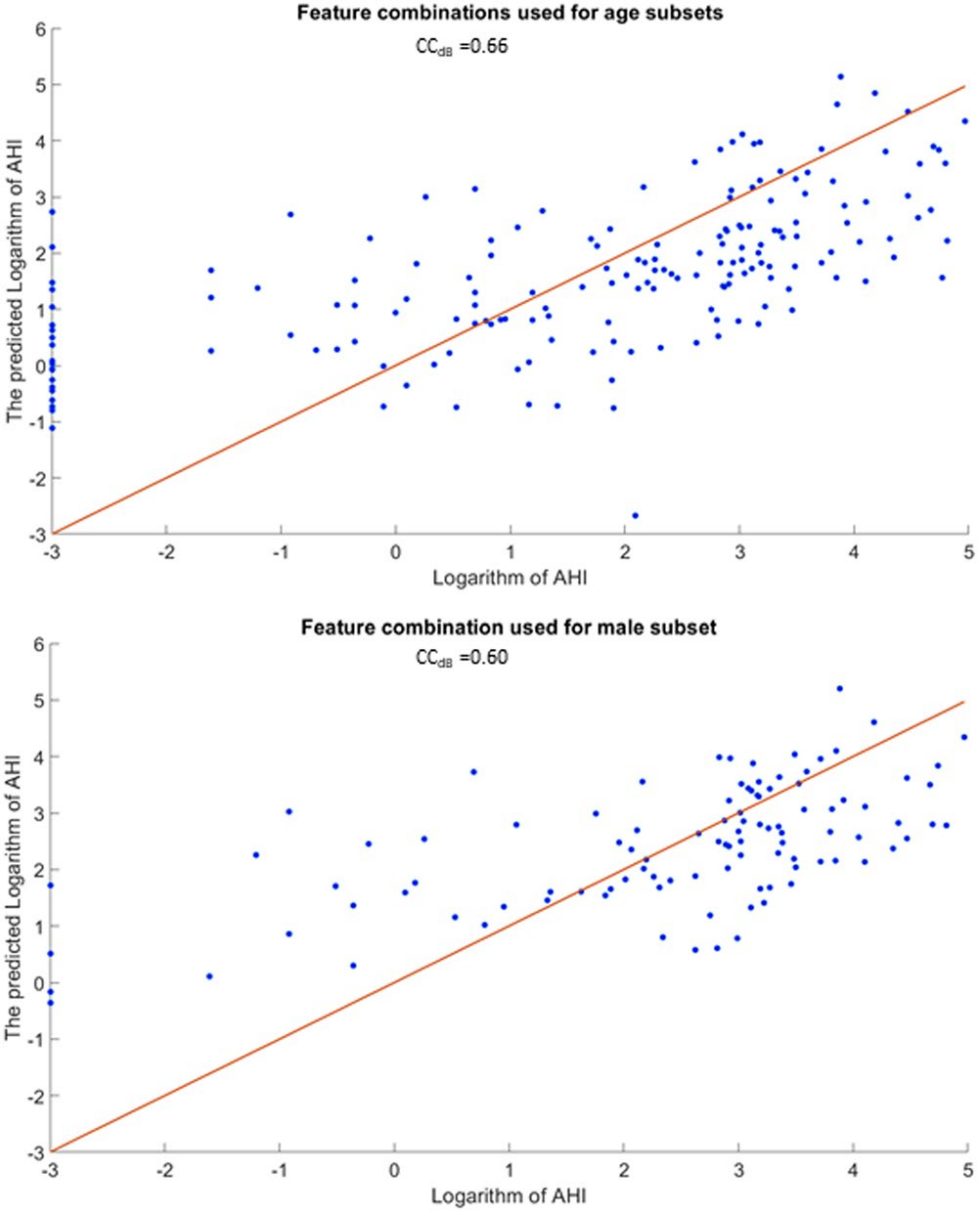
Linear regression models of feature combinations selected for age ≤ 50 (top) and male (bottom) subsets with the logarithm of AHI. Blue dots show the estimated logarithm of AHI values by the model. Legend: AHI: apnea-hypopnea index. CC: correlation coefficient.

Table 3 shows the classification accuracy, specificity, and sensitivity for the out of the bag-validation using Random Forest classification^27^ and for the blind testing dataset for each anthropometric subset separately as well as the final voted classification. In addition, Table 3 shows the correlation coefficient between AHI/logarithm(AHI) and each of the feature combinations using the linear regression analysis. Figure 2 shows the linear regression analysis outcomes of the selected feature combinations selected for age ≤ 50 and male subsets with the AHI in logarithmic scale. Furthermore, feature combination selected for age ≤ 50 showed the highest testing classification accuracy of 86% within its own subgroup. When this feature combination used for the entire training dataset and blind data set, it resulted in 71.6% accuracy for out of the bag-validation of the training set, and 75.6%, for the blind testing data.

**Table 3:** Correlation coefficient (Cc) of each feature combination and ahi and classification results using feature combinations for each anthropometric subset separately.

Groups	CC/CC_dB_	Out of bag-validation			Blind testing		
		Accuracy	Sensitivity	Specificity	Accuracy	Sensitivity	Specificity
BMI < 35	0.46/–	78.9%	81.8%	74.1%	68.4%	75.7%	55.0%
Age > 50	0.52/–	81.7%	83.3%	80.0%	67.4%	60.0%	73.9%
Age ≤ 50	0.63/0.66	85.7%	84.4%	87.5%	86.0%	85.2%	87.5%
Male	0.49/0.60	75.0%	71.9%	77.8%	64.6%	66.7%	63.3%
NC > 40	0.43/–	75.0%	75.0%	75.0%	64.7%	66.7%	63.6%
MpS ≤ 2	0.47/0.51	73.3%	71.7%	75.9%	74.6%	81.6%	64.0%
**Final voted results**							
Voted Accuracies	—	82.3%	81.4%	82.3%	**81.4%**	**82.1%**	**80.9%**

CCDB: CC with the logarithm of AHI, BMI: body mass index, NC: neck circumference, MPS: mallampati score.

**Figure 3:**
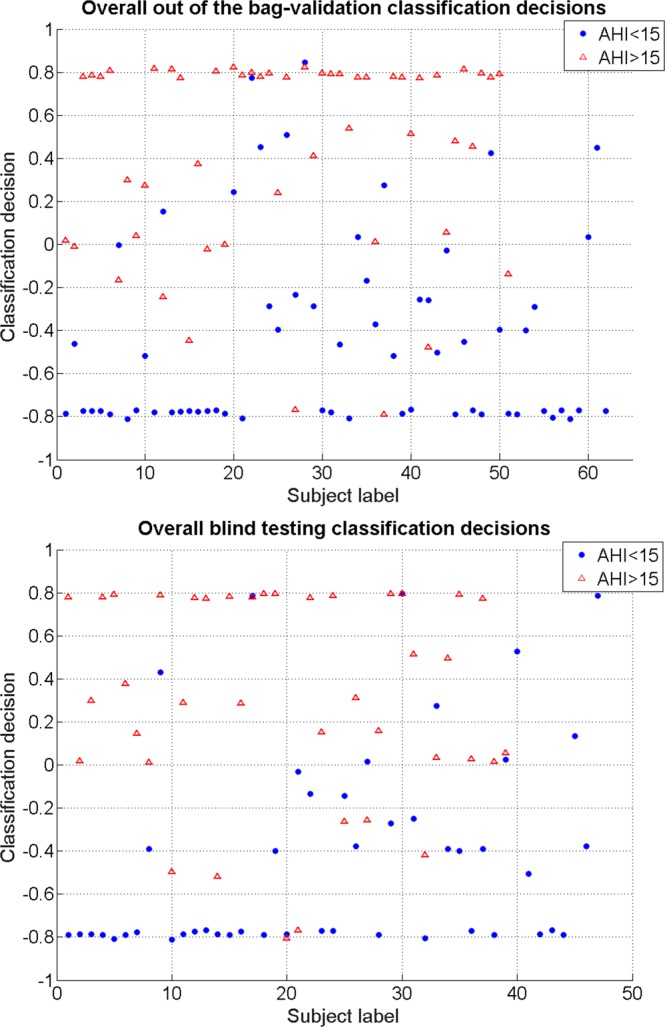
Scatter plot for out of the bag-validation in the training dataset (top) and blind testing (bottom) classification decisions; Blue and Red colors represent non-OSA and OSA individuals, respectively.

The overall classification results using the proposed weighted linear voting scheme, illustrated in Fig. 1, were found to be 82.3%, 82.3% and 81.4% for classification accuracy, specificity, and sensitivity for the out of the bag-validation, and 81.4%, 80.9% and 82.1% for classification accuracy, specificity, and sensitivity for the blind testing data, respectively. Figure 3 shows the scatter plot for overall out of bag-validation (top) and blind (bottom) testing classification decisions. A classification decision of 1 (considering 100% for both specificity and sensitivity) means that all the used random forest classifiers of each anthropometric subset voted the subject into the OSA group, while classification decision of −1 (considering 100% for both specificity and sensitivity) means all the used random forest classifiers of each anthropometric subset voted the subject into the non-OSA group.

Anthropometric information of the misclassified subjects in both out of bag-validation and blind testing classification are listed in Table 4. Out of 109 non-OSA subjects, 20 were misclassified to the OSA group, and out of 90 OSA subjects, 16 were misclassified to the non-OSA group. We also investigated whether removing a subset’s decision from the voting stage in the AWakeOSA algorithm would improve or degrade the overall classification results. The results indicated removing any of the subsets decreases the classification performance by 2.5–8%.

**Table 4:** Anthropometric information of all misclassified subjects within the training dataset (out of the bag-validation) and blind testing data.

	AHI	Age	Sex	BMI	NC	MpS
Non-OSA (AHI < 15, n = 20)	3.1 ± 3.1	45.9 ± 13.6	13 M, 7 F	36.7 ± 7.6	43.2 ± 3.4	13 ‘I’, 4 ‘II’, 2 ‘III’ and 1 ‘IV’
OSA (AHI > 15, n = 16)	37.0 ± 28.3	54.2 ± 11.8	13 M, 3 F	34.4 ± 6.9	43.3 ± 3.6	5 ‘I’, 6 ‘II’, 4 ‘III’ and 1 ‘IV’
Total (n = 36)	18.2 ± 25.3	49.6 ± 13.3	26 M, 10 F	35.7 ± 7.3	43.2 ± 3.4	18 ‘I’, 10 ‘II’, 6 ‘III’ and 2 ‘IV’

NC: neck circumference, BMI: body mass index, MPS: mallampati score.

## Discussion

Undiagnosed severe OSA significantly increases the healthcare cost and the risk of perioperative morbidity and mortality^8,9^. A quick, accurate and reliable screening tool can help resolve this. This study proposes a new algorithm (AWakeOSA) for screening OSA using anthropometric information and tracheal breathing sounds during wakefulness.

Anthropometric measures have been shown to have a high sensitivity in screening OSA^12,28^ but at the cost of a very poor specificity (~36%)^12^. This might be in part due to the subjectivism of most of the STOP-BANG parameters. Although those parameters do not have a good classification power to screen OSA, they are correlated with AHI and also affect breathing sounds^12,23–26^. In our dataset, Anthropometric parameters had a correlation of 0.03 < |r| ≤ 0.44 with AHI, and 0.00 < |r| ≤ 0.5 with breathing sounds. Previously, in our team, we showed that breathing sounds have a much higher classification power for screening OSA than the anthropometric features^16^. When we used only sound features for classification of OSA severity with a sharp AHI = 15, the classification accuracies were found to be 79.3% and 74.4% for out of bag-validation and blind testing, respectively. These accuracies are much higher than what STOP-Bang questionnaire can provide (63.4%) but not high enough nor sufficient for a reliable (robust) OSA screening during wakefulness.

In order to increase the accuracy and reliability of using breathing sounds to screen OSA during wakefulness, in this study, we propose a new algorithm, called AWakeOSA. This algorithm (shown schematically in Fig. 1) uses the anthropometric features to subgroup the data and get a classification vote in each subgroup, while the final classification is based on the weighted average vote of all subsets.; This subdivision has been done to reduce the impact of the confounding variables on the feature extraction stage and the classification process. The results showed that the new AWakeOSA voting algorithm provides a higher and more reliable blind test accuracy even with a sharp threshold of AHI = 15. The main challenge in all studies using breathing sounds analysis for wakefulness OSA screening is the heterogeneity of the population. As the cause of OSA can vary among individuals, people with the same OSA severity can have very different anthropometric features, and those differences affect respiratory sounds differently. The new proposed algorithm takes into account such heterogeneity and tries to find the best sound features specific to each subset of data that share one particular important confounding variable such as age, BMI, sex, etc. On the other hand, as the effect of these features on the sounds varies, we allow a weighting factor for each subset classifier’s vote. The weighting factor for each subset’s classifier vote is based the on sensitivity and specificity of the classifier developed in the training stage; please see the Appendix for details.

We divided the subsets based on age, sex, BMI, Mallampati score and neck circumference. Aging affects the female’s voice by decreasing its pitch, and the male’s voice by increasing its pitch^24^. Furthermore, aging causes muscle mass loss, drying of the mucous membrane, and increasing speech variability^25^; thus, we used two subsets for age > 50 and age < 50 separately. Sex was selected as a subset as it is known that females’ voice has a higher fundamental frequency pitch than men^23^, and that is independent of OSA severity; thus it is important to separately analyze males and females’ breathing sounds. BMI has a significant effect on the vocal quality and breathing sounds^26^; thus, we used two subsets for BMI > 35 and < 35. Mallampati score is an indicator of pharyngeal size, and therefore associated with narrowing of upper airway^29^; thus, MpS > 2 and <3 were chosen to form two subsets. The neck circumference is one of the most important OSA risk factors^30^; hence, we investigated participants with NC < 40 and >40 separately. If our dataset’s size was larger so that we could have more subjects in each subset, it might have been beneficial to form subsets based on height and weight independent of BMI as well. Nevertheless, BMI and NC are highly correlated with weight and height; thus, including subsets of weight and height may not improve the accuracy significantly.

Among the anthropometric subsets, the lower age subset (age ≤ 50) showed the highest testing classification accuracy (86%) compared to the others. This was not a surprising outcome. Age is a well-known risk factor for OSA^31^. Healthy individuals of age ≤ 50, in general, do not suffer from losing their upper airway muscle mass that in turn is responsible for upper airway collapse during an apnea/hypopnea event. They have more muscle tone than their age-matched OSA individuals. Thus, the loss of muscle tone is correlated with AHI in this group, and it affects breathing sounds significantly. In addition, after investigating the anthropometric information of the low age subset, we found that majority of non-OSA individuals in this subset also had BMI < 35, MpS < 3, NC < 40, and were females. On the other hand, the majority of the OSA individuals in this subset were from the opposite categories. Therefore, the low age subset was expected to have the highest classification accuracy among the other subsets to classify individuals with AHI > 15 and < 15.

Another interesting observation for the selected feature combination for low age and male subsets is their high correlation (0.66 and 0.6, respectively) with AHI in the logarithm scale as shown in Fig. 2. This implies the severity of OSA increases exponentially with an increase in AHI. Clinically, this is also implied as the challenge in OSA diagnosis for people with relatively low AHI. Otherwise, a very high AHI matches with apparent clinical symptoms.

**Figure 4:**
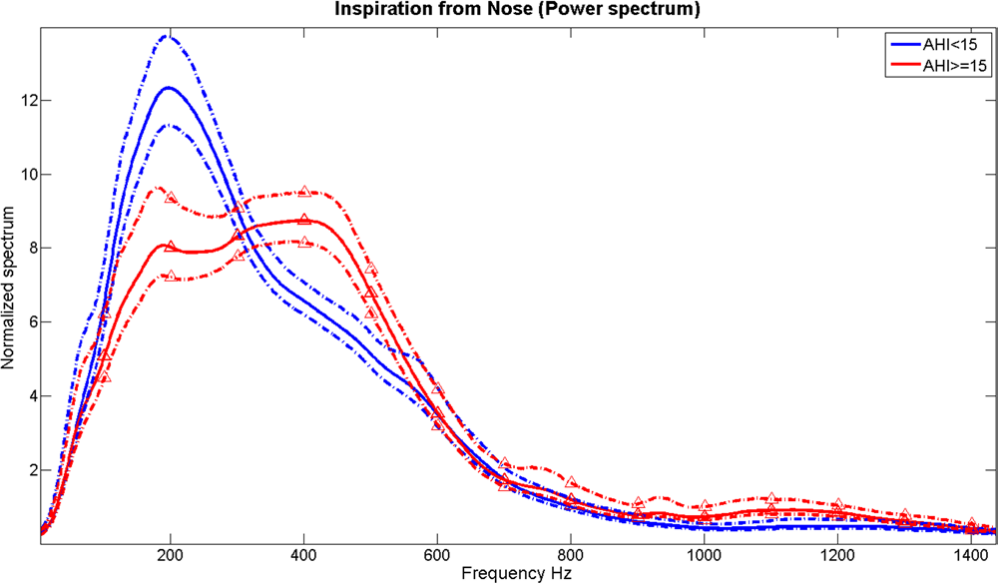
The average power spectrum of the signal recorded from nose inspiration. Dotted lines represent the 95% confidence interval. Red color represents the OSA group. Blue color represents the non-OSA group.

Among the selected sound features, only 6 features were extracted from frequency components above 1100 Hz, while the rest of the features were extracted from frequency components below 600 Hz. This indicates that OSA affects low-frequency components the most. Thus, it is important to record breathing sounds with devices that do not filter out either low or high frequencies. One of the best sound features has been the mean of the slope of the power spectrum of the nose inspiratory signal recorded within a frequency band of 250–350 Hz. This feature was selected 3 times in the following subsets age ≤ 50, Male, and MpS < 3. During nose inspiration, the upper airway’s muscles are active, while the pharynx cavity is patent. Pharynx cavity is responsible for transporting air from the nose to the lung, or the opposite. The upper airways of OSA individuals are commonly characterized by narrowing in the pharynx, thick tongue, losing muscles’ tone, thick and long soft palate^22^. These characteristics contribute to a significant narrowing of the upper airway cavity, increasing the chance of OSA during sleep. The mean of the spectral slope in 250–350 Hz shows that after 250 Hz the power of the sound of OSA individuals is increasing, but it is decreasing in non-OSA individuals (Fig. 4). It also shows the OSA individuals tend to have higher resonant frequencies than non-OSA individuals. These outcomes imply that OSA group is characterized by a more deformed and stiff upper airway than non-OSA. This is congruent with MRI/CT Imaging studies^21,22^ that showed the upper airway of OSA individuals during wakefulness on average had more regional compliance and stiffness.

Based on the final overall classification decisions (see Fig. 3), classifying a subject with an overall classification decision >0.7 or <−0.7 has about 90% confidence of being in the correct class. We also investigated whether all the subsets’ contribution to reach a reliable final vote for group assignment was significant. Excluding one of the selected subsets from the last voting stage degraded the overall classifier performance. Therefore, the proposed subsets are all critical to being considered in the analysis.

It is of interest to note the anthropometric parameters of the misclassified subjects. As can be seen in Table 4, the majority of misclassified subjects in the non-OSA group have age <50, **male-sex**, **BMI** **>** **35**, **NC** **>** **40** and MpS < 3. On the other hand, the majority of misclassified subjects in the OSA group have age > 50, male-sex, **BMI** **<** **35**, NC > 40 and **MpS** **<** **3**; bold words show risk factors for the opposite group based on the STOP-BANG questionnaire. This implies the anthropometric parameters or risk factors show correlations with AHI, yet do not have classification power and can result in misclassification.

### Limitations of the study

All participants were recruited randomly from those referred to the sleep lab for full PSG assessment. In this study, we only considered the anthropometric confounding variables as we did not have information about the plausible existing OSA comorbidities for our study subjects. A study has shown a high correlation of some comorbidity and OSA severity, and has suggested considering those comorbidity in determining OSA severity and its treatment options^7^. In this study, we did not have any information about the comorbid conditions of our study participants; the anthropometric information was the only available data along with the PSG reports. It is plausible that adding subgroups based on comorbidities would increase the accuracy of our AWakeOSA algorithm; that is a future goal of our studies.

Our dataset including 199 subjects has been the largest dataset studied on this topic; nevertheless, it is still not enough to overcome the heterogeneity of the population under study. We will need a much larger dataset so that we have an equal and reasonable number for every important anthropometric factor. For this study, we did not have enough numbers for the subgroups: females and individuals with NC < 40, MpS > 2 and BMI > 35. Sample size limitation also limits the Investigation of the reasons behind the misclassification. In addition, while our recorded sounds have had a large bandwidth compared to others in the literature, still it would be of interest to expand the bandwidth both at very low (down to 10 Hz) and high frequencies (up to 16 kHz) as they may reveal a better physiological interpretation.

## Conclusion

This study shows that anthropometric parameters affect the tracheal breathing sounds, and their effects can be positively utilized to improve the accuracy and reliability of OSA identification during wakefulness. All selected sound features for each anthropometric subgroup were statistically significant different (p-value < 0.05) between the two OSA groups. Despite using a sharp AHI threshold (AHI = 15), our proposed AWakeOSA algorithm shows a promising high classification blind-test accuracy with 82.1% sensitivity and 80.9% specificity. The AWakeOSA technology will be of great interest for OSA identification during wakefulness, in particular for anesthesiologists to be prepared accordingly for patients undergoing full anesthesia. As such reliable and quick OSA identification will reduce the perioperative resources and cost significantly. It will also help in reducing the number of undiagnosed OSA and the need for PSG assessment; thus, reducing the healthcare cost significantly.

## Methods

### Participants and Recording protocol

Study participants were recruited randomly from those referred to the overnight PSG assessment at Misericordia Health Center (Winnipeg, Canada). The study was approved by the Biomedical Research Ethics Board of the University of Manitoba. All experimental procedures were performed in accordance with the protocol approved by the Biomedical Research Ethics Board and its regulations. All participants signed an informed consent form prior to the experiments. The recording was performed about 1–2 hours prior to conducting the PSG study. During wakefulness and in the supine position (with head rested on a pillow), tracheal breathing sound signals were recorded using Sony microphone (ECM77B) embedded in a small chamber placed over the suprasternal notch of the trachea allowing ~2 mm space between the skin and the microphone. The participants were instructed to breathe deeply at the same flow rate, first, through their nose, and then, through their mouth with 5 breath cycles for each breathing maneuver. At the end of each breathing maneuver, participants were instructed to hold their breath for a few seconds to record the background noise (called silent period); for more details about the recording protocol see^16^. The AHI values were extracted from the PSG records from Misericordia Health Center after the overnight PSG assessment that was prepared by a sleep technician.

### Dataset

Out of the 300 recorded breathing sounds, data of 199 individuals were selected as valid data. The criterion to include an individual’s data was to have at least two clean (no artifacts, vocal noises, tones, interruptions, and low SNR) breathing cycles for each breathing maneuver. Each individual’s sound signals were inspected by audio and visual means in time and frequency domains to separate the inspiratory and the expiratory phases. At the time of our wakefulness recordings, we always marked the first inspiratory phase to help to achieve a 100% accurate separation of the two phases.

**Figure 5:**
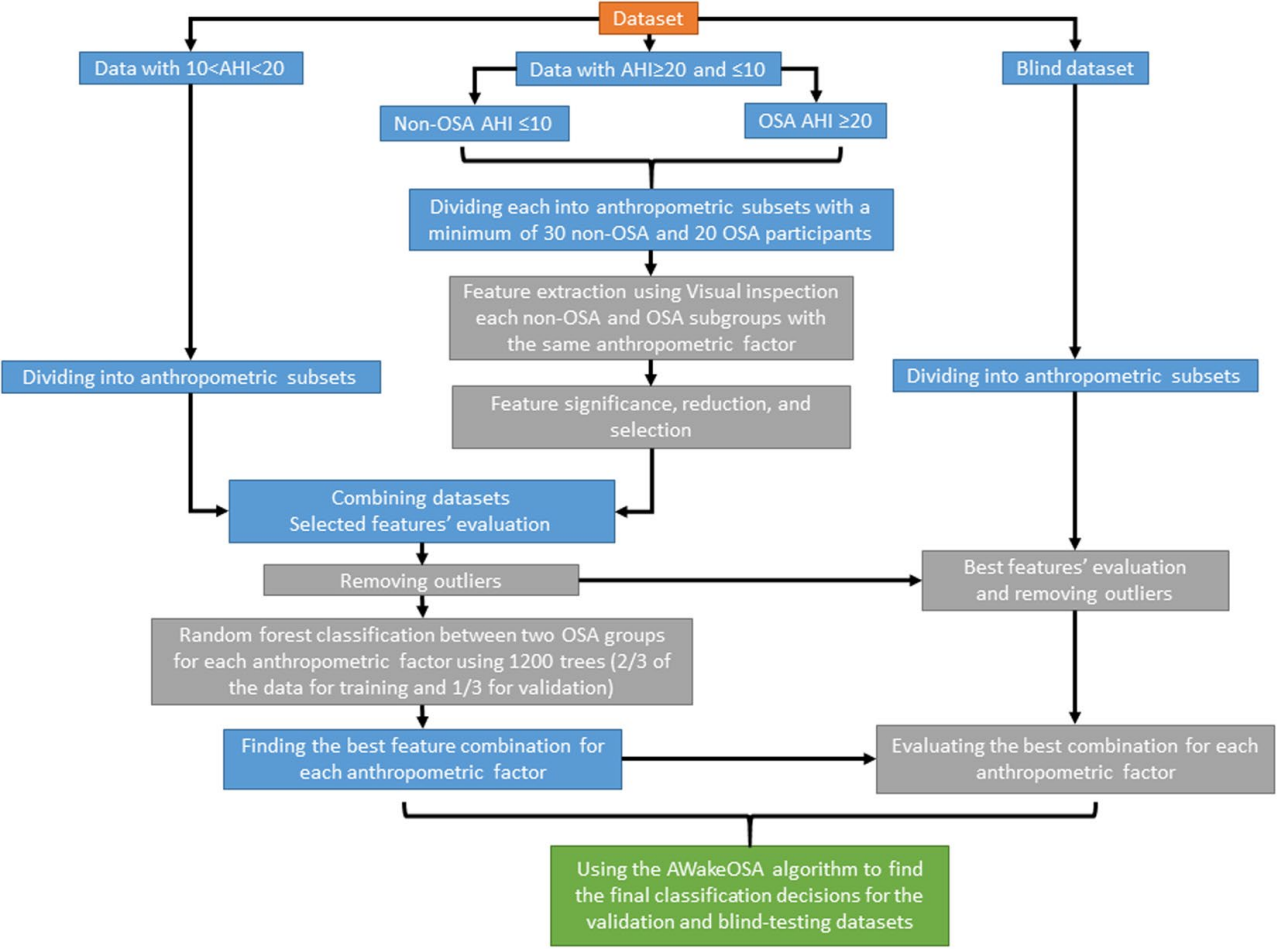
The workflow block diagram. AHI is apnea/hypopnea index.

This dataset included data of 109 individuals with AHI < 15 and 90 individuals with AHI > 15. For simplicity, we call these two groups as non-OSA and OSA groups, hereafter. A block diagram representing the methodology is presented in Fig. 5. Anthropometric information of the analyzed data (199 individuals) is presented in Table 1.

### Subsets’ Creation

Overall, data of 113 individuals were used for training and the remaining data of 86 individuals was used as the blind-test dataset. Within the training set, data of subjects with AHI ≤ 10 (n = 60) and AHI ≥ 20 (n = 40) were used for feature extraction for the two groups of the non-OSA and OSA, respectively; these data were from 100 subjects. Data of the remaining 13 subjects in the training dataset with 10 < AHI < 20 were not used for feature extraction but were included in the training classification process.

Within the dataset of the 100 subjects used for training, the following subsets were created: BMI < 35, Age ≤ 50 and > 50, Male, NC > 40, and MpS ≤ 2. These subsets had at least 30 and 20 individuals in each non-OSA and OSA groups, respectively.

### Signal analysis and Feature computation/extraction

We extracted spectral and bispectral features from the breathing sounds data. The signals power spectra were calculated using the Welch method^32^, and their bispectra using the indirect class of conventional bispectrum estimator^33^.

In our previous work^16^, we found that the frequency band could be divided into main four discriminative frequency bands (i.e., 100–300 Hz, 350–600 Hz, 1000–1700 Hz, and 2100–2400 Hz). Using these four frequency bands, the spectral and bi-spectral features were extracted. Some of the features (i.e., mean, standard deviation, spectral entropy, skewness and kurtosis, spectral centroid, etc.) were extracted from the non-overlapping area between the average spectra/bispectra and their 95% confidence intervals of the two groups of the training dataset. The minimum bandwidth to select a feature was set at 100 Hz. As an example, Fig. 4 shows the power spectra of the two groups’ inspiratory nose breathing.

We also calculated Katz and Higuchi fractal dimensions^34,35^, and Hurst exponent^36^ from the signals in the time domain. Therefore, for each subset, approximately, 250 features were extracted from the time and frequency domains analyses of the mouth and nose breathing sound signals. All features were scaled into the range of [0, 1].

### Feature reduction

#### Feature significance

We computed the p-value for each feature between the two OSA severity groups of the training dataset using the unpaired t-test. Any feature with a p-value > 0.05 was excluded.

#### Feature robustness

Each feature was assigned a robustness score. At first, all features had robustness scores of zero. Using the available individuals of the two severity groups, small groups of 15 per OSA severity group was created. These groups were randomly generated and shuffled until all the individuals were selected at least once. All combinations of the small groups generated for each severity group were created. For each feature and using each combination, the p-value and normality check for each small group of the two severity groups were computed, separately; Lilliefors test was used for normality check^37^. If the p-value was ≤0.05 and the normality check for each severity group was valid, the robustness score of this feature was increased by 1 point. This process was repeated 20 times. In the end, each feature had an overall robustness score. The features with an overall robustness score >0.6 of the maximum robustness score were selected for further analysis.

#### Feature correlation and redundancy

Using the available individuals’ data of the two severity groups, and using a support vector machine classifier, the training accuracy, specificity, and sensitivity were computed for each feature in each anthropometric subset of the training dataset. All correlation coefficients between any two features were computed. Any set of features with in-between correlation coefficient ≥0.9 were removed except the feature with the highest training classification accuracy, specificity, and sensitivity. The final set of features for each subset was selected by the end of this stage. We also checked the effect size of each of the selected features using Glass’s delta equation^38^.

### Training and validation

The selected features for each subset were evaluated using a total training data of 113 individuals (62 with AHI < 15, and 54 with AHI > 15) and a blind testing dataset of the 86 individuals (47 with AHI < 15, and 39 with AHI > 15).

#### Outliers’ removal

Using the training data for each OSA severity group of each subset separately, the outliers for each feature were removed, and the upper and lower adjacent values of boxplot were recorded^39,40^. Using the recorded values, the lowest lower adjacent value and the highest upper adjacent value were recorded. Using the blind testing data, any value outside the recorded boundaries was removed.

#### Feature combination creation and selection

Within each subset, the selected features were combined to create three-feature and four-feature combinations. Using each combination and the training data, a Random Forest^27^ classifier with 2/3 data in the bag (training) and 1/3 data out of the bag was used to evaluate the out-of-bag-validation testing accuracy, specificity, and sensitivity. We used the built-in function of Matlab^27^. The Random Forest routine included 1200 trees, interaction-curvature as a predictor selection, Gini’s diversity index as split-criterion. Cost matrix was used to compensate for the difference in the sample size between the two OSA severity groups. This procedure was repeated three times. Therefore, for each feature combination, it resulted in three values for each of accuracy, sensitivity, and specificity.

All values ≥0.7 were considered for the following stage, and the difference of the maximum and minimum of each three values were evaluated. The maximum difference for each of accuracy, sensitivity, and specificity was recorded. For each feature combination, the average value of each of accuracy, sensitivity, and specificity was recorded. The feature combinations with values between the maximum average and the difference between the maximum average and the maximum difference or 2% were selected to be the best feature combinations.

### Final combinations and blind testing

#### Each subset (separately)

Using the best feature combinations selected in the previous stage for each subset separately, and using Random Forest classifier with the same mentioned properties, the classification accuracies, sensitivities, and specificities of both the out of bag-validation and blind testing datasets were evaluated. This process was repeated 5 times; then, the average values were evaluated.

For each anthropometric subset, the feature combinations with the highest validation and blind testing accuracies were selected as the best feature combinations for that subset.

#### For the overall classification (last stage in the AWakeOSA algorithm)

The following process was done with and without including anthropometric features with the sound features. The overall classification was evaluated using feature combinations which each one was selected among the best for each subset. Different combinations were created due to having more than one final best feature combination per subset. The combination providing the highest overall classification accuracies, sensitivities, and specificities for both of out of bag-validation and blind testing datasets, was selected as the best feature combination.

The overall classification was conducted as follows:

1. Within each subset, the classification decision for each individual was evaluated; we assigned 1 and −1 labels to each class (OSA and non-OSA groups).
2. Using the outcomes of the subsets out of bag-validation, label 1 was multiplied by the sensitivity, and label −1 was multiplied by the specificity.
3. After conducting the previous two steps on all subsets, the weighted classification decisions of each individual were averaged to result in the final classification decision; any value > 0 or <0 was classified to OSA or non-OSA groups, respectively.

The misclassified individuals were investigated for any commonality between the OSA subjects misclassified to non-OSA, and/or the non-OSA subjects misclassified to OSA. The effect of neglecting a subset’s results in the overall classification process was investigated.

### Additional analyses

The correlation coefficient between AHI and anthropometric variables were investigated. Classifying the subjects using available variables of the STOP-BANG (i.e., Bang: BMI, age, NC and gender) were conducted, then the classification accuracy, sensitivity, and specificity were evaluated.

The correlation coefficient between AHI and the final selected sound features were investigated. Using the final selected feature combination for each subset, the correlation coefficients of the feature combination and AHI and its logarithm were evaluated.
